# Perceived Overqualification, Emotional Exhaustion, and Creativity: A Moderated-Mediation Model Based on Effort–Reward Imbalance Theory

**DOI:** 10.3390/ijerph182111367

**Published:** 2021-10-29

**Authors:** Zhanxue Gong, Fang Sun, Xiyuan Li

**Affiliations:** 1 School of Innovation and Entrepreneurship, South-Central University for Nationalities, Wuhan 430074, China; 2 School of Economics and Management, Wuhan University, Wuhan 430072, China; lixiyuan@whu.edu.cn

**Keywords:** perceived overqualification, emotional exhaustion, creativity, pay for performance

## Abstract

Overqualification is prevalent in times of economic downturn, and research has increasingly focused on its outcomes. This study aimed to explore the psychological burden caused by perceived overqualification (POQ) and its impact on creativity among high-tech enterprise employees. Drawing from effort–reward imbalance theory, we examined the effect of POQ on emotional exhaustion, along with the mediating role of emotional exhaustion in the POQ–creativity relationship and the moderating role of pay for performance (PFP) in strengthening the link between POQ and emotional exhaustion. Using cross-sectional data from a sample of 359 employees in China, we found that (1) POQ was positively related to emotional exhaustion; (2) emotional exhaustion was negatively related to creativity; (3) PFP moderated the effect of POQ on emotional exhaustion as well as the indirect effect of POQ on creativity via emotional exhaustion. These findings have both theoretical and practical implications.

## 1. Introduction

Even before the word pandemic re-entered the literary canon, more than one-third of the workforce felt that they had education, skills, and experience that far exceeded the requirement of their roles; namely, they were overqualified [1,2]. The global recession and a lack of job opportunities mean that more people feel overqualified for their position in recent years. The post-epidemic era will likely see some careers stagnate [3], and this phenomenon may have a tendency to spread. Overqualification has therefore attracted substantial academic and practitioner interest as a psychosocial risk factor in the workplace [4].

Both individuals and organizations are adversely affected by overqualification. In general, employees who feel overqualified tend to be less positive about their workplace [5], are more likely to be considering resignation [6], have a high propensity to be unproductive [7], and tend to be less healthy than others in the workforce [8]. However, there have been empirical studies that have shown the opposite is true and that perceived overqualification (POQ) can also lead to some good results, such as extra-role behavior [9], interpersonal altruism [10], and creativity and innovation [11]. The relationship between overqualification and creativity is important but has not been thoroughly researched [12], and consequently, it is ambiguous.

We focused on creativity, which involves generating new and useful ideas regarding products, services, practices, and procedures [13], as a negative reaction to POQ. Organizations today compete in a dynamic and uncertain environment in which creativity is highly valuable [14]. Creativity is believed to aid in decision making in ambiguous situations, such as those created by the COVID-19 pandemic, through improving flexible thinking and idea generation [15]. According to existing research, POQ and creativity are positively correlated. Due to their superior KSAOs (knowledge, skills, abilities, and other characteristics), overqualified workers can complete their contractual work at a much greater speed than their colleagues, and thus they can devote more of their time to think creatively [2,16]. In addition, overqualified individuals, as they possess surplus KSAOs, could carry out divergent thinking, association, and analogic reasoning, which are key to the process of creativity [17]. Moreover, academics state that motivating factors behind creativity could be the boredom [18,19] and job dissatisfaction [20] that accompany overqualification. Some researchers claim that POQ could have a positive impact on creativity, but only if contextual factors are taken into account [21,22]. However, there is still another possibility that the POQ–creativity link may be undesirable, as employees who perceive themselves as overqualified are considered lacking intrinsic motivation to participate in the creative process owing to poor person–job fit [21]. Accordingly, Lin et al. found that perceived underemployment had an inverted U-shaped relationship with creativity through the mediation effect of task crafting, which provides evidence for a potential negative relationship in this link [19].

Moreover, since the antecedents and conditions of creativity are complex and diverse, only limited studies have examined the mediation processes linking overqualification and creativity. There has been a great deal of research focused on cognitive and emotional variables as the process linking overqualification and its consequences [16]. The occupational stress levels of employees are believed to have a significant impact on creativity, organizational citizenship behavior, and counterproductive work behaviors [23], but no prior research has been conducted to examine the mechanisms between overqualification and creativity from this perspective. Effort–reward imbalance (ERI) theory states that overqualified employees put time and effort into acquiring KSAOs, which fails to result in a satisfactory social exchange through their work role [24]. This may lead to negative outcomes, such as emotional exhaustion and a reduction in creative outcome.

Research indicates that contextual factors may be a contributing factor to the inconsistent results of POQ [25]. According to Luksyte and Spitzmueller and the person–environment fit theory, contextual factors when looking at the POQ link to creativity. They looked at three of these factors: perceived organizational support, the mentoring of others, and idiosyncratic deals [21]. Zhang et al. uncovered the employee-development-oriented organizational cultures’ enhancement of the relationship of POQ and creative performance that was mediated by organizational identification [22]. Moreover, organizational identification was seen as another important boundary condition, such that the curvilinear interactive effect of perceived underemployment and organizational identification indirectly affects creativity through task crafting [26]. Schemes such as pay for performance (PFP) are beneficial because they provide recognition to overqualified employees based on the value they add, allowing practitioners to recognize overqualified employees more comprehensively [27]. Such schemes have become popular in recent years. Overqualified employees may feel less deprived and feel that they are being treated more fairly if they are paid according to their skills and competencies and not by the pay scale associated with their position [27]. This will go some way to prevent a lack of remuneration for the additional skills and competencies that overqualified employees possess, thus ensuring that the benefit of their human capital is given due consideration. The notion of using rewards to foster creativity has received attention in recent years [28]. One area that is interesting but has had limited research is the impact of organizational policies such as PFP and its effects.

We make several contributions to the literature with this study. First, drawing from ERI theory, we obtained a finding that differs from conventionally held mainstream beliefs that POQ has a negative impact on creativity. Our second contribution extends the scope of the existing research by testing the mediating role of emotional exhaustion in the impact of POQ on creativity. Finally, we established a boundary condition under which the negative effects of POQ would be aggravated or buffered by identifying PFP as a contextual moderator of critical work.

## 2. Theoretical Background and Hypothesis Development

### 2.1. Perceived Overqualification and Employee Creativity

POQ, which is an important dimension of underemployment, is defined as a feeling of having more experience, skills, and knowledge than is necessary for the job one is applying for or that one currently holds [5]. This phenomenon can be seen objectively from the perspective of an employer when comparing an applicant’s qualifications with the job description or requirements, or it can be looked at subjectively from the experience of an employee or a potential employee [1,29]. An overqualified individual’s subjective experiences are more likely to reveal the true complexities of that experience and to provide a more meaningful prediction of attitudes and behaviors in the workplace [30]. When considering the compatibility of employees’ qualifications with the job at hand, employees may evaluate their qualifications using different referents (e.g., past employment and current coworkers in similar positions). Overqualification may be experienced differently by two people in a comparable job [31], and as a result, their behavior is likely to be influenced by their perceptions in very different ways. Our consideration thus focused on an individual’s subjective perception of being overqualified.

Creativity is essential to sustained business success as it is necessary to recognize opportunities, anticipate unexpected changes, solve problems, adapt to technology, and create new businesses [32]. Individual creativity is the conceptualization of interactions in which employees provide novel and useful ideas regarding products, services, and processes at work that will be of benefit to multiple stakeholders [29,30,31,32]. Recent integrative reviews differentiated between creativity (an idea generation process) and innovation (i.e., the process of implementing ideas [33]). Consistent with this view, in our research, we focused on creativity as individual behaviors to generate novel and useful ideas, processes, and solutions [34]. Novelty reflects how a concept, an idea, or a product differs from traditional practices in a specific job or specific field; a given output is viewed as creative to the extent that appropriate observers independently agree that it is creative [35]. Usefulness refers to whether the ideas generated would benefit the organization; ideas that are not useful in enabling employees to do their jobs better are not considered creative [36,37]. Is there a reason why some people are more creative than others? To answer this question, a great deal of research has been conducted. Researchers have looked at a range of personal and contextual factors that might impact creatives, such as mood and personality [38], uncontrollability of the job [39], and organizational climates and team processes that provide creative behavior with support and autonomy [40,41]. Despite overqualification in the workplace being a widespread phenomenon and the acceptance that creativity helps maintain competitive advantages, it is surprising that there has not been much research in this area. Limited information is provided about whether, when, or how POQ impacts employee creativity. More than half of the researchers in this field have proposed theoretically that employees who feel overqualified could excel in fields requiring exceptional abilities, such as creativity, or in generating novel and useful ideas that would benefit multiple stakeholders [2,16,39,42]. However, only little empirical evidence has been provided to support that claim [21,22]. What is of interest is that, based on the job-crafting perspective, Lin et al. developed a serial curvilinear mediated-moderation model that relates underemployment to individual creativity and organizational citizenship behavior. They found that perceived underemployment and creativity through task crafting were interconnected through an inverted U-shaped relationship based on data collected from 327 teachers and their immediate supervisors. A moderating factor was organizational identification. This provides a possibility that overqualification and creativity may be negatively correlated. In addition, a lack of recognition and values and the insufficient nurturing of their skills and abilities are other consequences of overqualification. Without opportunities to express their confidence at work, individuals tend to develop a low level of self-efficacy [43], a negative psychological state, and a lack of motivation, all of which are important prerequisites for creativity. This also provides support for a potential negative relationship between the two. Consistent with this view, we intended to explore whether, how, and when perceived qualification has a negative effect on employee creativity.

### 2.2. Perceived Overqualification and Emotional Exhaustion

The ERI model in the workplace is built on the foundation of a general approach to analyzing psychological and social health and well-being. Ultimately, it claims that successful social exchange via salient roles contributes heavily to personal well-being and health in adulthood [24]. Social exchange plays a positive and negative role in personal self-regulation via marriage and parental roles, the work role, and various civic roles. Threats to this exchange, lack of reciprocity, or exclusion from this exchange weaken feelings of self-efficacy, self-esteem, and belonging (or self-integration), which have negative consequences for personal self-regulation. Human well-being seems to be dependent upon these three functions of self-regulation, and they reflect the delicate balance between a person’s self and social environment [24,44,45].

A work role is a great example in this regard as it offers options for all three functions of successful self-regulation: self-efficacy (e.g., good performance and personal growth and development), self-esteem (e.g., receiving recognition, sufficient compensation, the potential for promotion), and self-integration (social identity beyond the family, participation in social and workplace networks) [44,46]. Furthermore, earning a living is critical for determining the range of opportunities available in life. As an alternative, losing a job and being excluded from the labor market are two obvious examples of termination from a role that causes detrimental effects on health, self-regulation, and emotional well-being [47,48]. Losing a job is a stressful and visible experience within the workplace, but other experiences are widespread and potentially just as stressful. Arguably, one of these constellations of unfavorable social exchange could be a perceived lack of remuneration and reciprocity for the effort expended in the workplace.

In the case of overqualified employees, since they have further KSAOs and work experience than the work they acquire, the mismatch between the higher levels of KSAOs they have worked to achieve in the past and their current careers can be seen as a high-cost (e.g., education, skills, and experience)/low-gain (e.g., recognition, pay, responsibility) condition. Intentionally or unintentionally, employees may create high-cost/low-gain working conditions at work. The cost of disengagement from the labor market (e.g., the risk of being laid off or experiencing downward mobility) may outweigh the cost of accepting inadequate benefits, for instance, when other options in the labor market are not available [24]. This scenario is frequent for overqualified employees because of the fierce social competition, making it difficult for the employee to find a job that matches their qualifications. Consequently, they stay in unrewarding careers. In addition, people are willing to accept unfair job arrangements temporarily when they know it will increase their chances of career advancement and related rewards in their later career stage, which is also common for overqualified employees. Early in a professional career, for example, one may observe this pattern [49]. When a long-term investment in a career has failed, it can adversely affect a person’s self-regulation and cause them to experience stress, which plays a key role in reducing well-being and increasing illness susceptibility [49].

Our model focuses on emotional exhaustion as the underlying mechanism linking overqualification and employee creativity. As a prior dimension of burnout, emotional exhaustion refers to a chronic state of emotional and physical energy expenditure [50]. Stress-related fatigue, job-related depression, psychosomatic complaints, and anxiety are found in occupational stress research to be closely related to emotional exhaustion [51]. This observation indicates that emotional exhaustion can be reasonably theorized as a strain resulting from workplace stressors. Following the formulation of the original three-part model of burnout, the conceptual and empirical reasons for this can be identified. Drawing from the empirical evidence, emotional exhaustion has somewhat stronger relationships with outcome variables than the other components of burnout [52,53]. Conceptually, Shirom states that the core meaning of emotional exhaustion is the best way to define burnout [54]. In addition, academics have been able to differentiate burnout from other related concepts, such as self-efficacy and self-esteem, by emphasizing emotional exhaustion [54]. As a result of these empirical findings and conceptual frameworks, we focused on the emotional exhaustion aspect of burnout as the contributing factor.

There is a common belief that overqualified workers are less likely to be healthy and happy than adequately qualified employees, as well as empirical evidence to support that belief [29]. G.J. Johnson and W.R. Johnson surveyed postal workers, leading to their discovery that a significant negative correlation was found between POQ and employees’ subjective assessment of their general health, and a significant positive correlation was found between POQ and depression and stress symptoms [4,55]. Navarro, Mas, and Jiménez conducted a similar study using a sample of university professors. They found that POQ and fatigue were positively correlated [56]. Furthermore, the effort–reward ratio was found strongly associated with depersonalization and emotional exhaustion in a hospital study of 202 nurses in Germany. According to a significant interaction term between ratio e/r and overcommitment, nurses with a high level of overcommitment scored higher on both burnout scales [57,58]. Accordingly, here are the hypotheses:

**Hypothesis** **1** **(H1).** *POQ is positively related to emotional exhaustion.*

### 2.3. PFP as a Moderator

Through the construct of reward, social role agencies were posited to better understand the psychological costs of self-regulation failure [59,60,61]. ERI theory holds that role agency in social exchange is dependent on the experience of reward [24]. When this process is threatened, long-term stress may result leading to adverse work behavior. Research has established that a specific brain system plays a powerful role in motivation, reinforcement, and reward in personal and interpersonal well-being, the mesolimbic dopamine system, which innervates the prefrontal cortex, which is responsible for cognitive abilities and anticipation [24]. An inability to obtain anticipated gratifications or the frustration of reward expectations are likely to evoke sustained stress reactions in reward-sensitive neurons in the orbitofrontal cortex [24]. On the other hand, anticipation or achievement of rewards may reduce the negative impact of rewards. Psychological self-regulation reflects reward in emotions such as feelings of self-worth, belonging, and integration into an interpersonal, if not spiritual, context [24]. This can provide effective relief for negative feelings and stressful experiences.

Sociologically speaking, the reward is seen as a fundamental tenet of social exchange, the tenet of reciprocity and fairness: the exchange process is socially organized, and society contributes its rewards in return. It has been shown that sustained negative emotions can result from a violation of reciprocity in social roles [62]. Money, esteem, and career opportunities are distributed as scarce resources as rewards via three transmitter systems [24]. Organizations use compensation among other policy levers to motivate employee attraction, performance, and retention. An incentive plan that is based on employee performance is an organizational control method that motivates employees to perform in their specific roles by controlling their behaviors, outputs, or both [63]. Individual performance is typically a key factor in PFP at private companies in competitive markets, particularly as one moves from entry-level careers to higher levels [64,65,66]. PFP, regardless of the risks and challenges in its design and execution, is crucial for organizational effectiveness, according to current research [65]. Introducing PFP schemes that recognize overqualified employees according to their added value rather than according to their job pay scale may help them to feel less deprived and experience a more equitable level of compensation [27]. Therefore, overqualified employees can have their reward expectations fulfilled to a certain extent, as their additional skills and competencies are compensated adequately. As a result, the negative emotions and stressful experiences associated with the low-gain/high-cost status associated with POQ will be lessened. In sum, we suggest that PFP may affect the relationship between POQ and emotional exhaustion, i.e., when PFP is high, the negative relationship may be released.

**Hypothesis** **2** **(H2).** *PFP moderates the relationship between POQ and emotional exhaustion, such that the relationship is stronger when PFP is low rather than when it is high.*

### 2.4. The Mediated-Moderation Model

So far, we have determined that POQ has a positive impact on emotional exhaustion and that it is jointly influenced by levels of pay for performance. In our view, employee creativity is harmed by emotional exhaustion, and we expand on this view.

We predict that the lack of creativity can be attributed to emotional exhaustion for several reasons. As mentioned previously, emotional exhaustion is closely related to traditional stress reactions identified in occupational stress research [51]. Researchers have found a link between occupational stress and creativity. Literature discussing the relationship between psychological, organizational, and educational stress and creativity has drawn considerable attention [39]. Distraction arousal theory states that stress decreases creativity [67]. It is common for individuals to allocate some of their mental resources to stressors, with fewer resources remaining for other activities. As a consequence of reduced cognitive resources, simple cognitive strategies may also be used, such as narrowing attentional focus [68]. Common, less original ideas tend to be produced when cognitive strategies are simple [69]. We conclude that emotional exhaustion decreases creativity by depleting cognitive resources for creative thinking, leading to the use of simpler cognitive strategies that are likely to undermine creative thinking.

Second, even though a review of the burnout literature indicates that there is no clear link between emotional exhaustion and creativity, there is still much to be learned from the serious potential effects of burnout. The majority of researchers agree that burnout leads to a negative self-image [70], which undermines the three traditionally distinguished dimensions of creativity: fluency, flexibility, and original thought. Research has also shown that a high propensity to seek stimulation correlates positively with creativity [71]. When the demand for stimulation is reduced, this manifests itself as a slowdown of reactivity and blocks creativity in the early conceptualization stages. People who are burned out exhibit low levels of stimulation-seeking activity, which translates into high reactivity. There is a theoretical possibility that burnout can affect creativity in the intellectual and axiological domains. Burnout is also associated with absenteeism, turnover, and low morale. Self-reported measures of dysfunction, such as physical exhaustion, increased alcohol consumption, or other factors may be connected to burnout [69]. Burnout was described by Paine [72] as a pattern of personal distress that reduces mental capabilities and decreases professional performance. It causes rigidity and stifles flexibility and innovative thinking [73]. Storlie argued that burnout “is also resignation” [74]. Resignation suspends creativity and impairs the ability to make positive changes [74]. Furthermore, research that focuses on emotional exhaustion indicates that it can exacerbate and increase levels of anxiety in challenging situations [75], as well as increase levels of neuroticism. A person suffering from emotional exhaustion may also find it difficult to defend his or her point of view against external pressures. It also reduces one’s ability to act.

Although some scholars have suggested that burnout is not a synonym for a lack of creativity psychologically, hinting distinctly that the character of burnout is limited, the connection of this syndrome with creativity in an occupational area is still suggested [76]. These results provide impressive empirical evidence for the importance of emotional exhaustion in creative behavior since emotional exhaustion has emerged as a central variable for understanding burnout [77,78,79,80]. Our hypothesis, therefore, was that emotional exhaustion inhibits creativity.

**Hypothesis** **3** **(H3).** *Emotional exhaustion is negatively related to creativity.*

A performance-based pay policy alleviates some of the emotional exhaustion experienced by overqualified employees. A lack of creativity is a consequence of emotional exhaustion that harms organizations. Our previous theorizing and our current reasoning suggest that organizations can reduce the impacts of overqualified employees through appropriate compensation policies. Overqualified employees may feel less emotionally exhausted and regain creativity if PFP schemes recognize them for their added value rather than their pay scale. In this regard, the following hypothesis was proposed:

**Hypothesis** **4** **(H4).** *PFP moderates POQ’s indirect effect on creativity via emotional exhaustion, such that this indirect effect is more significant when PFP is low rather than when it is high.*

## 3. Method

### 3.1. Procedure and Sample

An employee survey of high-tech enterprises in China collected data for this study from employees across 26 provinces. Our participants were recruited by questionnaire websites, social media, and word of mouth. In the initial sample, there were 412 respondents. We excluded 52 respondents whose attention tests failed or whose data were significantly missing from our analyses (>12%), and this left a final sample of 359. The average age of the participants was 31.35 years old (SD = 4.45), and they had an average of 6.07 years of experience each (SD = 3.7). Of the 359 respondents, 192 (53.33%) were female. There were 23 who had a college degree or less (6.40%), 276 who had a bachelor’s degree (76.88%), and 60 respondents who had a master’s degree or higher (16.71%). The organizations where the respondents worked were mainly in high-tech industries.

### 3.2. Measures

Initially, the questionnaire was created in English but was administered in Chinese. Standard translation and back-to-back translation procedures were used to ensure measurement equivalence between the Chinese and English versions of the surveys [81]. Unless stated otherwise, each item was rated on a seven-point scale: one was “Strongly disagree” and seven was “Strongly agree”. Table 1 shows all the constructs and items in this study.

(1) Perceived Overqualification

Using Maynard, Joseph, and Maynard’s nine-item Scale of Perceived Overqualification, employees evaluated their perception of overqualification [82]. Items used for POQ were: “My job requires less education than I have”, “The work experience that I have is not necessary to be successful on this job”, “I have job skills that are not required for this job”, “Someone with less education than myself could perform well on my job”, “My previous training is not being fully utilized on this job”, “I have a lot of knowledge that I do not need in order to do my job”, “My education level is above the education level required by my job”, “Someone with less work experience than myself could do my job just as well”, and “I have more abilities than I need in order to do my job” (α = 0.94).

(2) Creativity

Creativity was measured using Madjar and Greenberg’s three-item scale for incremental creativity [83]. Items used for creativity were: “My project presents modification on previously existing work processes to suit current needs”, “I am good at using previously existing ideas or work in an appropriate new way”, and “I am very good at adapting already existing ideas” (α = 0.86).

(3) Pay for Performance

We used a three-item scale developed by Deckop et al. [84] to measure employees’ PFP. Items used for PFP were: “Increased productivity means higher pay for employees”, “My individual performance actually has little impact on any incentive pay award” (reverse-coded), and “My performance actually has little impact on my salary” (reverse-coded) (α = 0.81).

(4) Emotional Exhaustion

Watkins et al.’s three-item scale was used to measure emotional exhaustion [85]. These items included “I feel emotionally drained from my work”; “I feel burned out from my work”; and “I feel exhausted when I think about having to face another day on the job” (1 = never to 7 = very often) (α = 0.89).

### 3.3. Control Variables

The control variables in our study were gender, age, education, and tenure, in line with previous research [26]. The correlation between education and POQ has been shown in previous research on power distance cultures [2]. As a result, we controlled for education in all analyses, in addition to demographic variables. Control variables were coded as follows: Gender: “0” = female, “1” = male; Marriage: “0” = unmarried, “1” = married; Education: “0” = high school diploma or less, “1” = bachelor’s degree, “2” = postgraduate degree and above. It is worth highlighting that, to rule out the concern of potential p-hacking and increase the robustness of analyses, the data were reanalyzed without the control variables. We found that the results remained unaffected.

### 3.4. Analyses Strategy

Two stages were used in this study: confirmatory factor analysis (CFA) and structural equation modeling [86]. In general, the model fit is evaluated with four indices: the χ^2^ statistics, comparative fit index [87], normed fit index [88], and root mean square error of approximation [89]. 

Our team mean-centered both the independent variable (POQ) and the moderator (PFP) before analyzing data to avoid the potential of multicollinearity [90]. To test Hypotheses 1, 2, and 3, we used hierarchical multiple regression analyses. For the assessment of conditional indirect effects, Edwards and Lambert [91] suggested adding bias-corrected 95% bootstrapped confidence intervals. Therefore, we used PROCESS, a conditional process modeling program that uses ordinary least squares and logistic-based path analysis, to test Hypothesis 4. PROCESS tests for both direct and indirect effects [92]. An estimated conditional indirect effect was calculated using model 7 (using 5000 bootstrap samples).

## 4. Results

### 4.1. Common-Method Bias

To examine whether common-method bias was a concern, we controlled for the effects of an unmeasured latent method factor [93]. In this method, items are allowed to load on their theoretical constructs as well as onto a latent common-method variance factor [94]. When examining the item loadings with and without the common latent factor, none changed more than 0.15, which also suggests that common-method variance did not meaningfully affect our results [88,90].

### 4.2. Confirmatory Factor Analysis

Before testing our hypotheses, we investigated the convergent and discriminant validity of our focal constructs. We conducted a series of confirmatory factor analyses (CFAs) [86]. To test discriminant validity, CFA results in Table 2 showed that the four-factor measurement model (i.e., POQ, emotional exhaustion, PFP, and creativity) yielded a better model fit than the other alternative models, with χ^2^ =196.07, df = 71, NFI = 0.89, CFI = 0.93, and RMSEA = 0.07 (see Table 2) [95]. Thus, our study showed good distinctiveness of the four key variables. The discriminant validity of the model was confirmed.

Fornell and Larcker criterion results in Table 3 were used to evaluate the convergent validity of the measurements used in this study [96]. The average variance extracted (AVE), a measure of the variance captured by each construct [96], for satisfaction with POQ, creativity, PFP, and emotional exhaustion was 0.52, 0.68, 0.59, and 0.66, respectively, equal to or larger than the recommended cut-off value of 0.5 to establish convergent validity [96]. Composite construct reliabilities (CRs) were adequate, ranging from 0.85 to 0.87 [96]. Taken together, these results suggest that the four focal constructs in our study had good convergent validity. Overall, the four focal constructs in our study achieved both convergent and discriminant validity.

### 4.3. Descriptive Statistics

We present the means, standard deviations, and correlation of all variables in Table 4.

### 4.4. Hypothesis Testing

By individually entering the control variables and POQ in a hierarchical regression analysis, we tested Hypothesis 1 by considering emotional exhaustion as the dependent variable. As indicated in Model 2 of Table 5, POQ is positively associated with emotional exhaustion (β = 0.34, *p* < 0.01). Hypothesis 1 is therefore supported.

**Figure 1:**
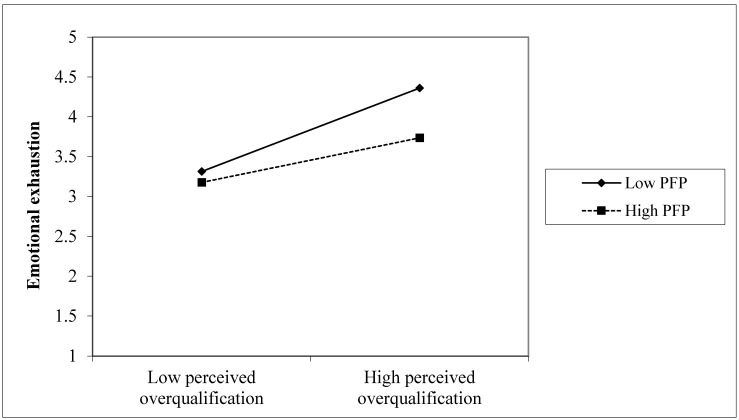
Moderating effect of PFP on the relationship between POQ and emotional exhaustion.

With respect to Hypothesis 2, we predicted that the positive relationship between POQ and emotional exhaustion would be weaker when employees’ PFP was high rather than lower. Results of hierarchical regression analysis reported in Table 5 indicate that the interaction term of POQ and PFP on emotional exhaustion was significant (β = −0.1, *p* < 0.05, Model 4). To better present the nature of the moderating effect, we plotted the interaction effect following the procedure outlined by Aiken and West [90] to operationalize high and low values of PFP using M ± 1 SD conditioning value. In support of our prediction, Figure 1 reveals that the slope of the positive relationship between POQ and emotional exhaustion was weaker for employees high in PFP (simple slope = 0.27, t = 3.35, *p* < 0.01), whereas the slope was stronger for employees low in PFP (simple slope = 0.51, t = 6.23, *p* < 0.01). As a result, Hypothesis 2 was confirmed.

As for Hypothesis 3, we predicted that emotional exhaustion is negatively related to creativity. The result indicates that emotional exhaustion significantly influences creativity (β = 0.26, *p* < 0.01, Model 7). This supports Hypothesis 3.

Hypothesis 4 predicted that PFP moderates POQ’s indirect effect on creativity via emotional exhaustion, such that this indirect effect is more significant when PFP is low rather than when it is high. Bootstrapping procedures were used to interrogate Hypothesis 4 and the moderated mediation model [91]. Table 6 reports the results of the conditional indirect effects at different values of pay for performance. It reveals that, at high levels of PFP, the indirect effect of POQ on creativity through emotional exhaustion was −0.16 and the 95 percent bias-corrected confidence interval did not contain zero (95% CI = [−0.12, −0.02]); at low levels of PFP, the indirect effect was −0.13 and the 95 percent bias-corrected confidence interval did not contain zero (95% CI = [−0.2, −0.07]). Furthermore, Table 6 also indicates that the difference in PFP in the conditional indirect effects of POQ on creativity through emotional exhaustion is significant (estimate = 0.07, SE = 0.03, 95% CI = [0.01, 0.14]). Concretely, the effect of POQ on creativity through emotional exhaustion is weaker when PFP is high rather than low. Hypothesis 4 is supported by these results.

## 5. Discussion

An analysis of a sample of employees in Chinese high-tech firms explored the mechanisms of influence between POQ and creativity. Our study was based both on the ERI theory and on the moderating role of PFP, an organizational policy. We concluded from cross-sectional data that POQ significantly reduces emotional exhaustion, which in turn decreases creativity. A further benefit of PFP is its capacity to attenuate the positive effect of POQ on emotional exhaustion, presumably through mediating its indirect impact on creativity through emotional exhaustion.

### 5.1. Theoretical Implications

In several ways, this study contributes to theory. To begin with, we studied creative talent from the standpoint of the ERI theory, a different approach from the mainstream view of previous research. Although current research focuses on exploring the positive outcomes of POQ, some researchers have called for more attention to be paid to the significant issue of POQ and creativity. Studies on this issue have generally shown a positive tendency, but empirical evidence has been scant. The study fills a gap to a certain degree by providing new evidence and possibilities regarding the impact of POQ on creativity, not only responding to demands for more research but also providing new possibilities for further study of this association.

Additionally, we utilized the ERI theory to include a new mechanism relating POQ to creativity by examining the mediating effect of emotional exhaustion. In accordance with ERI theory, we hypothesized that POQ, as a high-cost/low-gain condition in the employment relationship, causes employee emotional exhaustion, thereby damaging their creativity. By doing so, we advanced our understanding of the causal process linking POQ with creativity and provided a framework for understanding the relationship between POQ and employee exhaustion and creativity. Moreover, this study contributes to results in research related to the ERI theory. According to our research, employees do not only regard their abilities, time, and effort as inputs to a job but also consider that their qualifications, including education, skills, and experience, are a contribution to their employer. As a result, they should receive a reasonable return. Findings based on our research reveal that occupational stress occurs when individuals feel overqualified, which may further lead to higher levels of emotional exhaustion and a lack of creative thinking.

Thirdly, we presented a boundary condition at which the negative effects of POQ could be exacerbated or attenuated by identifying PFP as a contextual moderator of critical work. According to our findings, high levels of PFP can contribute to the emotional exhaustion linked to POQ. Our study of the moderating role of PFP provides a new and crucial boundary condition within which POQ can be detrimental to employees’ emotions and performance [97].

### 5.2. Managerial Implications

There are important implications for managers in our findings. A clear and effective compensation system can reduce the negative impact of the economic downturn caused by the impact of the epidemic on many employees by reducing the perception of overqualification. In designing a compensation system that is closely aligned with performance, employers can effectively reduce employees’ stress and negative emotions caused by this ERI. Additionally, to help employees maximize their creative performance, managers need to remove stressors that are likely to cause burnout in the workplace. It is also relevant that the selection process with which recruitment managers choose applicants should be closely scrutinized and should be designed to select the best candidates rather than overqualified applicants. A detailed job description should be provided by HR during the recruitment process so that applicants can gain a clear understanding of the requirements of the role.

### 5.3. Limitations and Future Research Directions

We had limitations in our study, as in most research. First, the main limitation of this study was the fact that it was cross-sectional. There are two major problems with this sort of study. First, the use of the job incumbent as the only source of data leaves many alternative explanations for observed correlations other than that the intended traits are related. Despite the fact that we performed the single-factor test that showed common-method bias was not a serious problem in this study, the results could have been affected by common-method bias [93] since the key variables were all collected at the same time and were self-reported. The second problem was that cross-sectional designs do not allow for confident causal conclusions. Caution is recommended in reaching conclusions concerning the causal relationships between the variables, as the current study did not capture causality variation [98]. Even the use of structural equation modeling cannot overcome the severe limitations of having all data collected concurrently. There are too many alternative explanations; for instance, the hypothesized link between POQ and creativity could be reasonably assumed to be the other way round: someone who is creative may also have higher work expectations and could be more vulnerable to POQ. As a result, we encourage researchers to use alternatives to self-reporting; specifically, constructs related to supervisor-reporting performance (i.e., creativity) are encouraged. In addition, a longitudinal design could be considered as an alternative to overcome this limitation in future research. Although, based on solid theoretical derivation, we proposed that it should be POQ that affects creativity, the reversed model is also an interesting and promising research direction for future studies.

Secondly, respondents were from a collectivist cultural setting as they were from China. The Chinese labor market has been in transition from being centrally planned to- ward being fully market-driven [99]. How individuals seek employment and how organizations manage their human resources in China are thus likely to bear more similarities with other market economies, such as the United States [100]. Given the export-oriented growth model in China and the significant mismatch between the education system and the needs of the labor market, underemployment is widespread in other than low-paying manufacturing and manual labor types of jobs [101]. As suggested in some estimates, 84% of Chinese employees currently feel overqualified for their jobs [102]. As such, underemployment is likely to be as prevalent in China as in Western countries. Moreover, as power distance is emphasized and harmonious interpersonal relationships are valued [95] in the Chinese context, POQ may not be accepted as widely in China as it is in other countries. Employees with collectivist attitudes are less likely to perceive themselves as overqualified; they are also more tolerant of POQ, according to Hu et al. [12]. Thus, we could have underestimated the effects of overqualification on creativity as the prevalence of overqualification among Chinese employees. We urge researchers to conduct more research in diverse cultural contexts.

## Figures and Tables

**Table 1:** Constructs and items.

Constructs	Items	Sources
POQ (Perceived overqualification)	My job requires less education than I have.	[82]
	The work experience that I have is not necessary to be successful on this job.	
	I have job skills that are not required for this job.	
	Someone with less education than myself could perform well on my job.	
	My previous training is not being fully utilized on this job.	
	I have a lot of knowledge that I do not need in order to do my job.	
	My education level is above the education level required by my job.	
	Someone with less work experience than myself could do my job just as well.	
	I have more abilities than I need in order to do my job.	
Creativity	My project presents modification on previously existing work processes to suit current needs.	[83]
	I am good at using previously existing ideas or work in an appropriate new way.	
	I am very good at adapting already existing ideas.	
PFP (Pay for Performance)	Increased productivity means higher pay for employees.	[84]
	My individual performance actually has little impact on any incentive pay award.	
	My performance actually has little impact on my salary.	
Emotional exhaustion	I feel emotionally drained from my work.	[85]
	I feel burned out from my work.	
	I feel exhausted when I think about having to face another day on the job.	

**Table 2:** Results of the confirmatory factor analysis.

Model	X2	df	X2/df	NFI	CFI	RMSEA	Δχ^2^	Δdf
Four-factor model	196.07	71	2.76	0.89	0.93	0.07		
Three-factor model 1	457.49	74	6.18	0.74	0.77	0.12	261.41	3
Three-factor model 2	729.29	74	9.86	0.59	0.61	0.16	533.22	3
Three-factor model 3	379.66	74	5.13	0.79	0.82	0.11	183.59	3
Two-factor model	644.43	76	8.48	0.64	0.66	0.14	448.36	5
One-factor model	942.07	77	12.24	0.47	0.49	0.17	745.99	6

One-factor model: POQ, emotional exhaustion, PFP, and creativity were combined into one factor. Two-factor model: creativity and PFP were combined into one factor; emotional exhaustion and POQ were combined into another. Three-factor model 1: POQ and emotional exhaustion were combined into one factor. Three-factor model 2: PFP and emotional exhaustion were combined into one factor. Three-factor model 3: PFP and creativity were combined into one factor.

**Table 3:** Reliability and validity of scales.

Variable	Cronbach’s α	CR	AVE
POQ	0.94	0.84	0.52
Creativity	0.86	0.87	0.68
PFP	0.81	0.81	0.59
Emotional exhaustion	0.89	0.85	0.66

Note: CR = composite reliability, AVE = average variance extracted.

**Table 4:** Means, standard deviations, reliabilities, and correlations among study variables.

	M	SD	1	2	3	4
1. POQ	3.59	1.02	1			
2. PFP	5.16	1.16	−0.16 **	1		
3. Emotional Exhaustion	2.77	1.27	0.39 **	−0.22 **	1	
4. Creativity	5.50	0.87	−0.15 **	0.13 *	−0.32 **	1

N = 359. * *p* < 0.05; ** *p* < 0.01.

**Table 5:** Results of hierarchical regression analysis.

	Emotional Exhaustion				Creativity			
	M1	M2	M3	M4	M5	M6	M7	M8
Controlled variable								
Age	−0.05	−0.03	−0.03	−0.04	0.05	0.04	0.04	0.04
Tenure	−0.04	−0.05	−0.05	−0.05	0.03	0.04	0.02	0.02
Gender	−0.11 *	−0.09	−0.12	−0.11 *	0.08	0.07	0.05	0.05
Marriage	−0.18 **	−0.16 **	−0.15 **	−0.14 **	0.15 *	0.14 *	0.1	0.1
Education	0.08	0.03	0.05	0.03	0.01	0.03	0.03	0.03
Independent variable								
POQ		0.34 ***	0.31 ***	0.32 ***		−0.11 *		−0.03
Mediator								
Emotional exhaustion							−0.26 ***	−0.25 ***
Moderator								
PFP			−0.16 **	−0.15 **				
Interaction								
POQ×PFP				−0.1 *				
F	5.31 ***	12.94 ***	12.89 ***	11.91 ***	3.21 **	3.44 **	6.95 ***	5.98 ***
ΔF	5.31 ***	47.59 ***	10.52 ***	4.19 *	3.21 **	4.45 *	24.61 ***	20.1 ***
R^2^	0.07	0.18	0.21	0.21	0.04	0.06	0.11	0.11
ΔR^2^	0.07	0.11	0.02	0.01	0.04	0.01	0.06	0.05

N = 359. * *p* < 0.05; ** *p* < 0.01; *** *p* < 0.001.

**Table 6:** Conditional indirect effects of POQ on creativity through emotional exhaustion at levels of PFP = M + 1 SD.

Values of PFP	Conditional Indirect Effect	SE	95%CL	
			Lower	Upper
Low PFP	−0.13	0.03	−0.2	−0.07
High PFP	−0.06	0.03	−0.12	−0.02
Differences	0.07	0.03	0.01	0.14

Note. Results are based on 5000 bootstrap samples.

## Data Availability

The data presented in this study are available on request from the corresponding author. The data are not publicly available due to respondents’ privacy.
